# Good and Bad Stroma in Pancreatic Cancer: Relevance of Functional States of Cancer-Associated Fibroblasts

**DOI:** 10.3390/cancers14143315

**Published:** 2022-07-07

**Authors:** Ryota Ando, Akihiro Sakai, Tadashi Iida, Kunio Kataoka, Yasuyuki Mizutani, Atsushi Enomoto

**Affiliations:** 1 Department of Pathology, Nagoya University Graduate School of Medicine, Nagoya 466-8550, Japan; ando.ryota@med.nagoya-u.ac.jp (R.A.); sakai.akihiro@med.nagoya-u.ac.jp (A.S.); iidatyuw@med.nagoya-u.ac.jp (T.I.); kkataoka@med.nagoya-u.ac.jp (K.K.); y-mizu@med.nagoya-u.ac.jp (Y.M.); 2 Department of Gastroenterology and Hepatology, Nagoya University Graduate School of Medicine, Nagoya 466-8550, Japan

**Keywords:** pancreatic cancer, cancer-associated fibroblasts, stroma, tumor microenvironment, Meflin, immunoglobulin superfamily containing leucine-rich repeat, fibrosis

## Abstract

**Simple Summary:**

Recent progress in research on the biology of cancer-associated fibroblasts (CAFs) in pancreatic ductal adenocarcinoma (PDAC) indicates their diverse states and plasticity, which may lead to good and bad stroma, suppressing and promoting cancer progression, respectively. The characteristics of the stroma differ spatially, even within the same tumors, based on the balance between cancer-restraining CAF and cancer-promoting CAF proliferation at the site. These heterogeneous CAFs also influence the sensitivity of PDAC to anticancer therapeutics. Further preclinical and clinical studies will advance our understanding of the roles of CAFs in disease progression and aid the development of therapeutics that modulate or ameliorate the tumor microenvironment in PDAC.

**Abstract:**

A well-known feature of human pancreatic ductal adenocarcinoma (PDAC) is the extensive proliferation of cancer-associated fibroblasts (CAFs) and highly fibrotic stroma. Recent evidence, based mainly on single-cell analyses, has identified various subsets of CAFs in PDAC mouse models. However, we do not know how these CAF subsets are involved in the progression and drug resistance of human PDAC. Additionally, it remains unclear whether these diverse CAFs have distinct origins and are indicators of genuinely distinct CAF lineages or reflect different states of the same CAFs depending on the tumor microenvironment. Interestingly, recent preclinical studies have started to characterize the nature of cancer-restraining CAFs and have identified their markers Meflin and collagen type I alpha 1. These studies have led to the development of strategies to induce changes in CAF phenotypes using chemical reagents or recombinant viruses, and some of them have been tested in clinical studies. These strategies have the unique potential to convert the so-called bad stroma to good stroma and may also have therapeutic implications for non-cancer diseases such as fibrotic diseases. Together with recently developed sophisticated strategies that specifically target distinct CAF subsets via adoptive cell transfer therapy, vaccination, and antibody–drug conjugates, any future findings arising from these clinical efforts may expand our understanding of the significance of CAF diversity in human PDAC.

## 1. Overview of Pancreatic Cancer-Associated Fibroblasts

**Figure 1:**
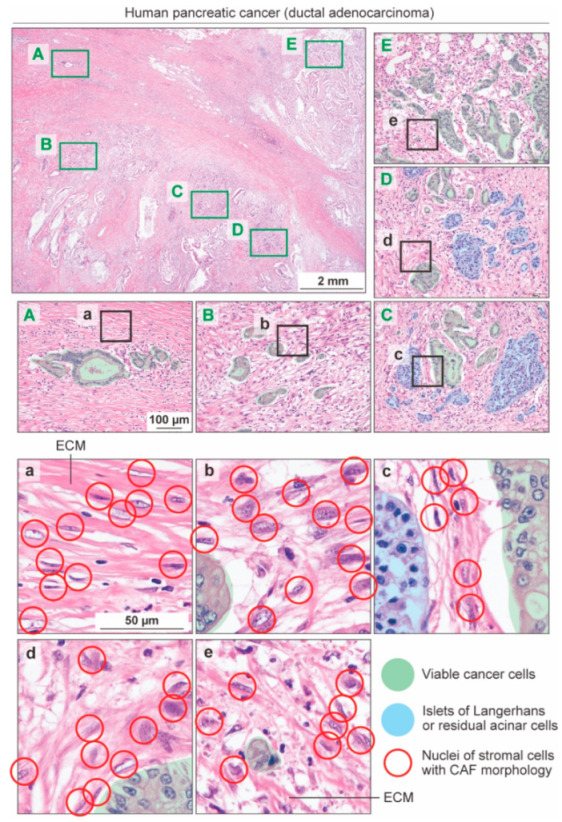
A representative histology of human pancreatic cancer. A low-magnification image of a tissue section of human pancreatic ductal adenocarcinoma (PDAC) stained with hematoxylin and eosin is shown in the top left panel. The areas outlined in green (**A**–**E**) are magnified in the adjacent right and lower panels. The areas outlined in black (**a**–**e**) are magnified in the lower panels with the same magnification. The circles represent stromal cells with CAF morphology. Viable cancer cell nests and islets of Langerhans/acinar cells are shaded in green and blue, respectively. ECM, extracellular matrix.

Cancer-associated fibroblasts (CAFs) are among the major components of the tumor microenvironment (TME) [1,2,3,4,5,6,7]. CAF proliferation is conspicuous, particularly in intractable and aggressive cancers such as pancreatic ductal adenocarcinoma (PDAC) (Figure 1) [8,9]. Ever since the significance of fibroblasts in the progression of prostate cancer was first demonstrated using a mouse model [10], CAFs have attracted considerable attention from researchers. Subsequently, the notion that CAFs promote cancer progression and could therefore be a target for the development of new anti-cancer therapeutics has become widely accepted [1,2,3,4,5,6,7].

One major function of CAFs is the production of extensive amounts of extracellular matrix (ECM) proteins, such as collagen and fibronectin [1,2,3,4,5,6,7,11,12]. These proteins are produced by CAFs deposited in the stroma, leading to changes in the mechanical properties of tumor tissues, such as stiffness, interstitial pressure, and collagen configuration [13]. CAFs also produce several growth factors, cytokines, and chemokines, many of which promote cancer cell proliferation and motility [1,2,3,4,5,6,7]. These liquid factors subsequently attract and recruit many types of immune cells, including lymphocytes and myeloid cells, to tumor sites, thereby suppressing anti-tumor immunity [14]. Furthermore, CAFs produce various proteases that degrade and remodel the ECM to help cancer cell groups invade the stroma effectively and collectively [15,16,17,18,19]. They are eventually activated downstream of many growth factors produced by cancer cells [1,2,3,4,5,6,7,20]. Thus, CAFs are generally considered to be promoters or accelerators of cancer progression. There is ample evidence to prove that cancer cells co-opt preexisting normal fibroblasts to become CAFs and shape the TME, which facilitates cancer progression [1,2,3]. Many published review articles focus on describing the cells of origin, activating mechanisms, and functions of CAFs, which readers should refer to in order to understand the current review better [1,2,3,4,5,6,7].

**Figure 2:**
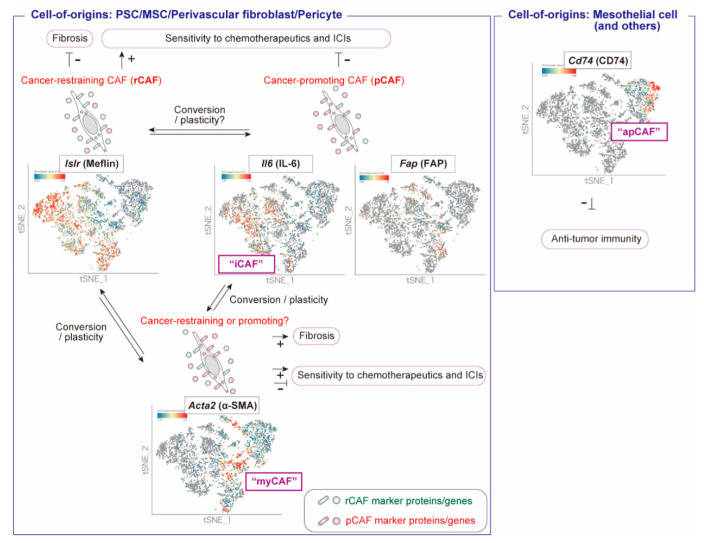
Current classification of CAFs in PDAC. The expression of the indicated CAF marker genes (*Islr*, *Il6*, *Fap*, *Acta2*, and *Cd74*) in CAFs isolated from tumors of the KPC mouse model of PDAC was visualized using t-distributed stochastic neighbor embedding (t-SNE) plots. Publicly available single-cell RNA sequence data of all fibroblasts isolated from four KPC mouse with PDAC tumors (GEO accession code: GSE129455, Elyada et al. [24]) were analyzed and visualized using the Bioturing Bbrowser. It should be noted that different CAF marker genes were expressed in different CAFs at varying degrees of overlap. Recent studies have shown that Meflin protein encoded by the *Islr* gene possesses cancer-suppressing roles; however, the precise roles of Meflin^+^ CAFs have not been demonstrated clearly. α-SMA^+^ myCAFs play a role in suppressing the progression of pancreatic cancer in mice; however, the functions of α-SMA protein and other proteins expressed in myCAFs have not yet been demonstrated. apCAFs are derived from mesothelial cells and have a different origin from other types of CAFs.

The morphological diversity of CAFs in human PDAC can be better appreciated by the close histological examination of tissue sections stained with conventional hematoxylin and eosin (H&E) (Figure 1). By moving the field of view by several millimeters, one can easily recognize that the morphology, nucleus and cell sizes, and density of CAFs, as well as the patterns of ECM deposition, are completely different between the lesions, even within the same patient (Figure 1a–e). Accordingly, advanced technologies for single-cell transcriptomic analysis have clearly shown that CAFs are molecularly heterogeneous, or diverse in terms of gene expression (Figure 2) [4,5,21]. Several different CAF classifications based on different clustering methods and differing focal points for research have been summarized in many review articles and will not be repeated in this review [4,5,21]. The most established CAF classification in PDAC was proposed by Tuveson et al., who categorized CAFs into three subsets: myofibroblastic CAFs (myCAFs), which robustly express α-smooth muscle actin (α-SMA); inflammatory CAFs (iCAFs), which produce inflammatory cytokines such as interleukin (IL)-6; and antigen-presenting CAFs (apCAFs), which have recently displayed the potential to induce regulatory T cells and suppress anti-tumor immunity [22,23,24] (Figure 2). One of the unaddressed issues, however, is that we still do not know whether these different CAFs are indicators of genuinely distinct CAF lineages, or if the same CAFs reflect different states depending on the changes in the TME; an exception is apCAFs, which are derived from mesothelial cells [25]. Another issue with these CAF classifications seems to be that the biological significance of the given CAF marker genes or the given CAF subsets has not been demonstrated clearly, mainly because of the lack of specific markers for the given CAF subsets, and genetically engineered mouse models that enable us to specifically deplete or ablate the given CAF subsets. The clinical relevance of these CAF subsets in human PDAC has not yet been fully elucidated.

As an alternative method of CAF classification, we considered a simple CAF classification which categorizes CAFs based on whether they are pro- or anti-tumorigenic [1,2,26,27] (Figure 2). This classification was originally proposed by Kalluri and his colleagues and assumes the existence of cancer-promoting CAFs (pCAFs) or tumor-promoting CAFs (TP-CAFs) and cancer-restraining CAFs (rCAFs) or tumor-restraining CAFs (TR-CAFs), and the balance between these CAFs is crucial for cancer progression, the regulation of anti-tumor immunity, and sensitivity to anti-cancer therapeutics [2,5]. In recent years, our group has focused on understanding the nature of rCAFs, and the data obtained so far imply that the nature of rCAFs may be similar to that of tissue-resident fibroblasts, mesenchymal stem cells (MSCs), or pancreatic stellate cells (PSCs) [28,29]. Interestingly, this balance between rCAFs and pCAFs can be modulated by therapeutic reagents, as shown through a pioneering study by Sherman et al., who first showed that the vitamin D analog calcipotriol changes the phenotype of CAFs, thereby improving tumor sensitivity to chemotherapeutics in a PDAC mouse model [30]. In this short review, we summarize recent studies on rCAFs in PDAC and the relevance of therapeutic strategies which convert pCAFs into rCAFs or modulate their balance. We speculate that the diversity and complexity of CAFs could be demonstrated through a simple model wherein CAFs exist on an overlapping functional spectrum from anti-tumor rCAFs to pro-tumor pCAFs, which constitute the “good” and “bad” stroma, respectively, of human PDAC.

## 2. Current Understanding about rCAFs in PDAC

Rhim et al. [31], Özdemir et al. [32], and Lee et al. [33] first reported that all or some CAFs have tumor-suppressive functions in PDAC. They also showed that suppression of CAF proliferation, either through genetic depletion of sonic hedgehog (SHH) in cancer cells, use of SHH inhibitors, or genetic ablation of α-SMA^+^ CAFs, resulted in the progression of PDAC in mouse models. These data raised the hitherto unconsidered question of whether some, but possibly not all, populations of CAFs play an inhibitory role in cancer progression in mice, and α-SMA is a candidate marker of those CAFs [32]. Based on these findings, researchers have hypothesized that CAFs can be categorized into two functionally distinct subsets: pCAFs and rCAFs [2]. In contrast, other studies have suggested the involvement of TGF-β, a potent cytokine that induces α-SMA expression in fibroblasts and stromal fibrosis, and its downstream signaling pathways in the progression of pancreatic cancer in the advanced stages [34]. In mechanistic terms, another study showed that the RNA interference-mediated depletion of the α-SMA gene led to a significant decrease in the nuclear accumulation of the mechanosensitive transcription factor YAP/TAZ in cultured MSCs, which are known to be one of the origins of CAFs [35]. YAP/TAZ expression in CAFs is crucial for cancer cell invasion and ECM stiffening [36]. Therefore, it seems appropriate to argue that the α-SMA protein expressed in CAFs may enhance the malignant features of cancer; however, its precise in vivo roles remain undetermined. Taken as a whole, the most reasonable interpretation of previous studies is that α-SMA^+^ CAFs suppress cancer progression, whereas the role of α-SMA protein remains elusive in in vivo contexts, including human cancers (Figure 2). The Kaplan–Meier analysis is not considered helpful in determining the functions of the genes of interest in terms of whether they are pro-tumorigenic or anti-tumorigenic. For example, the expression of an rCAF marker gene may be high in aggressive tumor cases with desmoplastic and fibroinflammatory reactions, because they are heavily infiltrated with both rCAFs and pCAFs. Those stromal reactions are often associated with poor outcomes, which does not necessarily mean that the product encoded by the CAF marker gene promotes tumor progression.

Recent studies have gone a step further in determining the role of α-SMA^+^ CAFs. Researchers have engineered a genetically modified mouse model in which the gene encoding collagen type I alpha 1 (Col1α1) was specifically depleted in α-SMA^+^ cells (Col1^smaKO^ mouse) [37]. When crossed with autochthonous PDAC model mice, Col1^smaKO^ mice developed pancreatic tumors that were more aggressive and exhibited poorer outcomes than the control mice did; this observation was associated with the recruitment of myeloid-derived suppressor cells and the suppression of CD8^+^ cytotoxic T cells. These data showed that Col1α1 is a protein that functionally defines rCAFs in PDAC.

Our group recently focused on another protein, Meflin, which is specifically expressed in CAFs in both human and mouse PDAC [26,28,29,38]. It is a glycosyl-phosphatidylinositol-anchored protein encoded by the gene immunoglobulin superfamily containing leucine-rich repeats (*ISLR*), which is also secreted into media containing cultured fibroblasts [29,39]. To date, two proteins have been identified as Meflin-interacting proteins. One protein is bone morphogenetic protein (BMP) 7, which is a cytokine that functionally counteracts the pro-fibrotic function of TGF-β and restrains fibrosis in multiple organs [40,41,42]. Meflin binds with BMP7 to augment its downstream activation of Smad1/5, thereby inducing the expression of inhibitors of DNA binding proteins Id2 and Id3. Another Meflin ligand is lysyl oxidase (Lox), which is a crosslinker of collagen fibers that promotes fibrosis and tissue stiffening [43,44,45,46]. Our biochemical analysis showed that Meflin interacts with Lox to inhibit its collagen cross-linking activity [43]. Consistent with this result, the Meflin expression levels in CAFs are anti-correlated with straighter and wider collagen structures in PDAC mouse models. Furthermore, Meflin deficiency in CAFs is associated with a more aggressive histology of PDAC and resistance to chemotherapeutics, suggesting that Meflin may be a functional marker of rCAFs in PDAC [28,43]. Our study showed that Meflin exhibits cancer-restraining activity; however, the precise role of Meflin^+^ CAFs has not been demonstrated clearly.

Functional rCAF marker proteins other than Col1α1 and Meflin may exist. Some studies have shown that decorin, a matricellular protein that has multiple functions, including the modulation of receptor tyrosine kinase signaling, is expressed in CAF subsets and plays a role in suppressing cancer progression [47,48]. According to a previous study, the expression of nerve growth factor receptor (NGFR, also known as CD271) in CAF is correlated with a favorable prognosis in patients with PDAC [49]. We speculate that future studies will further identify the functional rCAF marker proteins. Most importantly, no master transcription factor(s) that determines the identity of rCAFs has been identified to date.

## 3. States of CAFs, Not CAF Subsets, May Be Responsible for “Good” or “Bad” Stroma in Human PDAC

**Figure 3:**
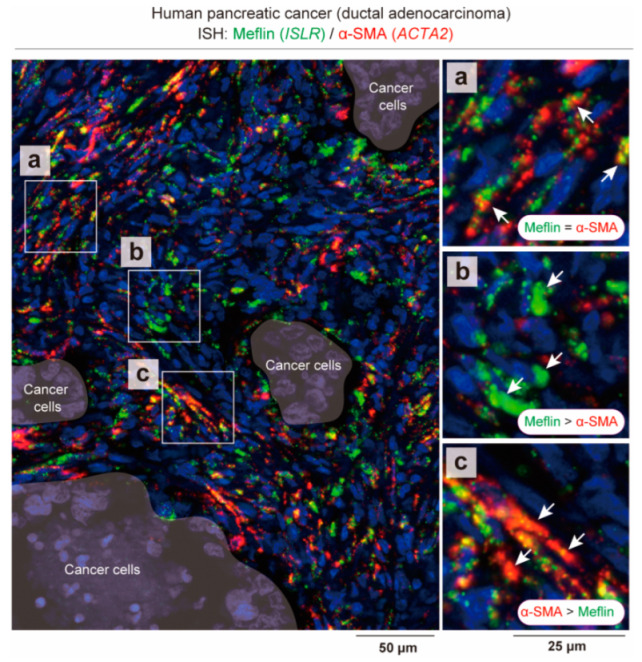
Overlap and continuity in the expression of genes encoding Meflin and α-SMA between different CAFs. Duplex in situ hybridization (ISH) assay shows an overlapping but inverse correlation between Meflin (*ISLR*; green) and α-SMA (*ACTA2*; red) expression in CAFs infiltrating human PDAC. The boxed areas (**a**–**c**) are magnified in adjacent panels. The arrows indicate CAFs that exhibit variable levels of Meflin and α-SMA expression. Statistical analysis of the inverse correlation between Meflin and α-SMA expression in CAFs was performed in Mizutani et al. [28].

It is difficult to distinguish between CAF subsets based on gene expression because no markers are specific to any CAF subset and their gene expression profiles overlap with each other. As is evident from single-cell transcriptomic analyses, highly specific and sensitive multiplex in situ hybridization (ISH) assays show that CAFs positive for Meflin (*ISLR*) mRNA are also positive for α-SMA (*ACTA2*) mRNA to varying degrees (Figure 3) [28]. Notably, Meflin and α-SMA mRNA expression levels are inversely correlated with each other: CAFs that are highly positive for Meflin mRNA are weakly positive for α-SMA mRNA, whereas those weakly positive for Meflin mRNA are highly positive for α-SMA mRNA (Figure 3) [28]. According to our research, this continuity in gene expression between different CAFs is better demonstrated using ISH, which detects the mRNA expression level, rather than immunostaining, which detects the protein level. This proved true when we investigated the relationship between α-SMA and matrix-remodeling-associated 8 (MXRA8), a new CAF-specific marker which is co-expressed with Meflin in both human and mouse PDAC [50].

**Figure 4:**
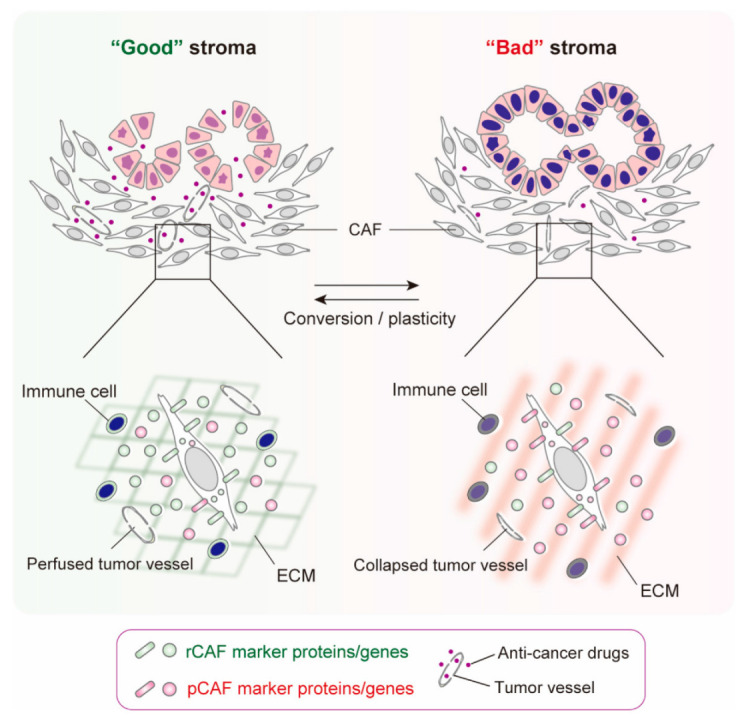
Hypothesis regarding the states of CAFs that determine the properties of the stroma of PDAC. The state of individual CAFs may be regulated by the balance between the expression of rCAF and pCAF marker proteins, which results in the formation of good (left) or bad (right) PDAC stroma. Good and bad stromata affect the properties of tumor vessels and immune cells, which determine the progression and drug sensitivity of PDAC. Recent studies have suggested that rCAFs and pCAFs are plastic and can be converted into each other during disease progression and pharmacological interventions [21,43].

These observations led us to hypothesize that, other than apCAFs, which are derived from mesothelial cells [25], CAFs cannot be clearly separated into subsets with distinct cells of origins and functions (Figure 2). Nevertheless, they are skewed or polarized to any one type of CAFs, and diverse CAFs reflect different “states” of the same CAF, depending on the TME (Figure 4). Given the heterogeneous features of human PDAC, as revealed by conventional H&E staining (Figure 1), it is plausible that the TME of PDAC is composed of varying quantities of good stroma, which is rich in CAFs that express rCAF markers, and bad stroma, which is rich in CAFs that express pCAF markers, and the net balance between the good and bad stroma determines the progression, drug sensitivity, and tumor immunity of human PDAC (Figure 4). The significance of the balance between rCAFs and pCAFs has also been shown in mouse and human colorectal cancer. The ratio of Meflin^+^ rCAFs and pCAFs that are positive for Gremlin 1, a potent BMP antagonist, is crucial for the outcome of patients with colorectal cancer, and the exogenous manipulation of the balance between Meflin^+^ rCAFs and Gremlin 1^+^ pCAFs improves liver metastasis in colorectal cancer [41].

As extensively reviewed in other review articles on CAFs, the state of CAFs, or whether a stroma is good or bad, may be associated with the tumor response to immune checkpoint inhibitors (ICIs), and there is accumulating evidence that CAF phenotypes are involved in anti-tumor immunity, which will not be reiterated here [1,2,3,4,5,51,52]. We recently showed that the number of Meflin^+^ rCAFs is associated with a favorable objective response rate in patients with non-small-cell lung cancer treated with ICIs [53]. Again, this observation seems to contradict the prevailing notion that CAFs generally contribute to tumor resistance to ICIs [54,55]. A recent study showed that IL-6 production from α-SMA^+^ myCAFs, but not pCAFs, marked by the expression of fibroblast activation protein-α (FAP-α), contributes to the resistance of pancreatic cancer to chemotherapy and ICIs (Figure 2) [56]. Thus, the rCAF and pCAF balance seems to be important for regulating antitumor immunity and sensitivity to ICIs, although currently no ICIs have been shown to be effective in the treatment of human PDAC.

## 4. CAF Conversion and Plasticity

The regulatory mechanisms of the conversion between rCAFs and pCAFs have begun to be elucidated [1,2,21]. The stimulation of cultured fibroblasts with TGF-β was shown to induce the rapid downregulation of Meflin expression, whereas α-SMA was significantly upregulated [40,41]. Fibroblasts cultured on stiff substrates, such as the plastic dishes generally used in laboratories, also induce Meflin downregulation and α-SMA upregulation [40]. Meflin is expressed in undifferentiated MSCs, and its expression is downregulated upon their differentiation into adipo-, osteo-, and chondrogenic cell lineages and myofibroblasts [57,58]. Other factors that downregulate Meflin expression include hypoxic conditions, microgravity, and continuous cell passage on plastics [29,40,59]. Consistent with these study findings, the lineage tracing of Meflin^+^ CAFs revealed that they give rise to α-SMA^+^ CAFs during PDAC progression in mice, supporting the speculation that rCAFs convert into α-SMA^+^ CAFs, which may contribute to CAF heterogeneity [28]. In contrast, Col1α1 expression is downregulated by a soft substrate, but the detailed mechanisms by which Col1α1 expression is regulated in rCAFs are not well known [35,60]. Col1α1 expression was recently examined in a breast cancer cell line and found to be upregulated by the transcription factor MRTF-A, on which the TGF-β, Wnt/β-catenin, and Rho/Rho-kinase signaling pathways converge [61]. Thus, the transcription of the same rCAF marker proteins, namely, Meflin and Col1α1, is differentially regulated, which may complicate our understanding of the nature of rCAFs. Although not described in detail in this review, there is strong evidence of the involvement of epigenetic regulation in altering the CAF phenotype [1,2]. Therefore, it is reasonable to speculate that the conversion of rCAFs into pCAFs is also regulated by histone modification and DNA methylation.

We recently showed that exogenous delivery of Meflin through a recombinant Sendai virus vector into tumors that were developed in a subcutaneous transplantation model of PDAC induced an increase and decrease in the numbers of Meflin^+^ and α-SMA^+^ CAFs, respectively [43]. Thus, tumor sensitivity to the chemotherapeutic agent gemcitabine was improved. This observation suggests that the CAF phenotype can be genetically manipulated, which may help in developing a therapeutic strategy in the future.

Another strategy that has been recently developed is the pharmacological conversion of the CAF phenotype using chemical reagents [21]. As described above, Shermann et al. showed that activated PSCs, which are almost equivalent to CAFs, are reprogrammed into quiescent PSCs upon treatment with the vitamin D analog calcipotriol [30]. The administration of calcipotriol to an autochthonous PDAC mouse model (KPC model) resulted in the decreased expression of several iCAF and myCAF markers, such as IL-6 and α-SMA, accompanied by the increased tumor vascular area and chemosensitivity of the developed tumors. Thus, the study by Sherman et al. showed that calcipotriol has the potential to revert pCAFs to rCAFs or quiescent PSCs. PSCs are resident stromal cells of the pancreas which are characterized by an abundance of vitamin A in their cytoplasm [62]. Consistent with this study finding, the administration of all-trans retinoic acid (ATRA) exerts an effect similar to that of vitamin D in reverting activated PSCs to quiescent PSCs and consequently suppressing the progression of PDAC in mice [63,64]. Based on these observations, multiple clinical trials investigating the efficacy of the combination of calcipotriol or ATRA with conventional chemotherapeutics or ICIs in patients with PDAC are underway [21].

Our group recently discovered that Am80, a non-natural synthetic retinoid, effectively upregulates the expression of Meflin in CAFs from PDAC mice, suggesting that Am80 may have the potential to convert Meflin-negative or weakly positive pCAFs into Meflin-positive rCAFs [43]. Consistent with the antifibrotic role of Meflin, enacted by augmenting BMP7 signaling and suppressing Lox activity, the oral administration of Am80 induced changes in collagen configuration, decreased tumor tissue stiffness, and increased tumor vessel area. These effects were accompanied by an increase in drug delivery efficiency and chemosensitivity in the PDAC mouse model. Interestingly, a comparison of genes that were differentially expressed between Am80- and calcipotriol-treated CAFs revealed that Meflin expression was more significantly upregulated by Am80 than by calcipotriol [43]. Our preliminary experiments on a subcutaneous transplantation PDAC mouse model showed that the oral administration of Am80 significantly improved gemcitabine efficacy compared to the intraperitoneal administration of calcipotriol (T.I. and A.E., unpublished observation). Based on these findings, our institution started a clinical study that investigated the safety and efficacy of the combination of Am80 and conventional chemotherapeutics, such as gemcitabine and nab-paclitaxel, in patients with advanced unresectable PDAC [65].

Another strategy for converting CAFs was suggested in a recent study which showed that targeting Pin1, which is a proline isomerase involved in multiple oncogenic pathways, significantly attenuated the expression of α-SMA in myCAFs as well as of inflammatory cytokines (IL-6, LIF, CXCL12) in iCAFs in the tumor stroma of a PDAC mouse model, which was accompanied by a significant increase in the sensitivity of tumors to chemotherapeutics [66].

## 5. Conclusions

In this short review focusing on rCAFs in PDAC, we describe our current understanding of the nature and identity of rCAFs and their phenotypic conversion and plasticity during disease progression. We believe that different “states” of CAFs may exist in human PDAC and influence the formation of good or bad stroma, depending on the TME and disease stages (Figure 4). Good stroma may be mainly composed of rCAFs, normal tumor vessels, and immune cells with higher anti-tumor activity, whereas bad stroma may be composed of pCAFs, structurally and functionally abnormal tumor vessels, and immune cells with lower anti-tumor activity, and the net balance of these compartments may determine the progression and drug sensitivity of PDAC. However, the master regulators which orchestrate CAF states are not completely understood. The roles of rCAFs in rare types of cancer remain elusive [67,68]. Interestingly, Meflin^+^ fibroblasts have been found in the stroma of non-tumor fibrotic diseases, such as cardiac fibrosis, idiopathic pulmonary fibrosis, and kidney fibrosis, where they seem to be essential for tissue repair, but also play a role in suppressing fibrosis, unlike α-SMA^+^ myofibroblasts, which promote tissue fibrosis and stiffening [40,69,70]. These findings suggest common mechanisms and etiologies of cancer and fibrotic diseases. Thus, further understanding of the biology of rCAFs could be helpful for the development of therapeuti not only for cancer, but also for fibroinflammatory diseases.
